# Development status of young new farmers from the perspective of vocational ability: A research analysis based on 21 cities in Guangdong Province

**DOI:** 10.1371/journal.pone.0349224

**Published:** 2026-05-22

**Authors:** Tao Luo, Qingpeng Huang, Jingyan Mai

**Affiliations:** 1 Youth League Committee, Guangdong Mechanical and Electrical Polytechnic, Guangzhou, China; 2 School of Automotive, Guangdong Mechanical and Electrical Polytechnic, Guangzhou, China; Alexandru Ioan Cuza University: Universitatea Alexandru Ioan Cuza, ROMANIA

## Abstract

Professional competence refers to the comprehensive qualities that an individual needs to possess in order to continuously develop and achieve excellent performance in their professional activities. As more and more young people enter the agricultural sector, the professional competence of young new farmers not only affects their personal growth but also directly influences the transformation of agriculture and the development of rural areas. Based on data obtained from in-depth interviews and questionnaires, this paper examines the career development of young new farmers from three aspects: entering agriculture, rooting in agriculture, and developing agriculture, with a focus on their identity formation, influencing factors, and development paths. Research shows that the professional abilities of young new farmers mainly include three levels: professional skills, general abilities, and core literacy. The improvement of abilities at different levels requires corresponding cultivation methods. The research conclusions can provide references for the training of young agricultural talents and the improvement of rural talent revitalization policies.

## 1. Introduction

### 1.1 Research background

Implementing the rural revitalization strategy and promoting the modernization of agriculture and rural areas are key to achieving China’s long-term development goals. Talent development is the primary driving force behind rural revitalization, and young agricultural professionals play a crucial role in the process of agricultural modernization [1,2]. According to employment statistics, the proportion of bachelor’s degree holders working in the primary sector has increased significantly, rising from 0.7% in 2017 to 1.0% in 2021, marking a 43% increase [3].

In addition, by the end of the 13th Five-Year Plan, returnee entrepreneurs and incoming village entrepreneurs have become an important part of the new type farmers, with their number reaching 10.1 million, and it is expected to exceed 15 million by 2025 [3]. Although an increasing number of young talents are entering the agricultural field, they still face many obstacles in skill development, resource acquisition, and career growth, and the topic of professional sustainability remains at the forefront. Therefore, professional competence has become an important factor that influences their professional development and the sustainable transformation in agriculture [4].

Professional competence is primarily reflected in areas such as specialized knowledge, practical skills, and adaptability, and serves as a key foundation for young new farmers to achieve long-term career development. As the rural revitalization strategy continues to advance, the importance of this group in agricultural transformation and rural development is steadily increasing [5,6]. The current research mainly focuses on specific regions or groups, which leads to an insufficient understanding of the overall professional capabilities of young new farmers. Most existing studies focus primarily on theoretical discussions or case analyses, and there is a lack of systematic empirical research based on samples from different regions and with diverse disciplinary backgrounds. To make up for this deficiency, this study analyzed the professional abilities, influencing factors, and improvement paths of young new type farmers through questionnaires and in-depth interviews. The research, from a cross-regional and cross-disciplinary perspective, comprehensively presents the professional roles and development characteristics of young new farmers in modern agriculture.

### 1.2 Research status

#### (1) Research on vocational ability.

Research on vocational competencies currently focuses primarily on two areas: first, the dimensions that make up vocational competencies; and second, the assessment and application of vocational competencies. In terms of the dimensional composition of professional competence, many scholars have proposed different structural classifications. For instance, Jiang [7] summarizes professional competence as a combination of professional ability, methodological ability, social ability, and practical ability; Li [8] on the other hand, adopts a hierarchical approach, categorizing these into core employment competencies, industry-specific competencies, and occupation-specific competencies.

With regard to the assessment of professional competencies, scholars have proposed a variety of measurement tools. For example, the Kasseler competency classification model is used to examine innovative personality traits in occupational contexts [9], and the Learning Potential Assessment Center (LP-AC) evaluates an individual’s leadership potential by simulating work situations [10]. In addition, tools such as ASCOT, SOLO, and COMET have been used to measure vocational competencies in a more systematic manner [11–13]. However, most of the current assessment methods are not specifically tailored for the new type of agricultural workforce, and thus are unable to fully reflect their occupational characteristics. Therefore, it is still necessary to construct a more industry-specific analytical framework.

#### (2) Research on new farmers.

Since 2013, studies on “new farmers” have been increasing, but there is still no consensus on the definition of this term. The current research mainly understands this group from three perspectives: Firstly, from the perspective of the internet, it emphasizes how they utilize digital technologies, e-commerce platforms and social media to transform the agricultural production and marketing methods [14,15]. Second, from the perspective of the industrial chain, it is believed that it is not only engaged in production but also participates in brand building, marketing, and technical services [16]. Third, from the perspective of business transformation, it emphasizes the introduction of modern management concepts into various sectors of agriculture to drive the upgrading of the agricultural value chain [17].

Based on the above arguments, some scholars have further expanded the definition of “new farmers.” They believe that this group should possess market awareness and technical skills, as well as the basic capabilities required for modern agricultural production and operation. They hold a crucial position in the agricultural value chain and actively participate in the rural transformation process by applying new technologies, enhancing production efficiency, and promoting rural economic development [18,19]. New farmers contribute significantly to industrial development, injecting new vitality into rural communities through initiatives such as promoting ecological agriculture, improving rural governance, and revitalizing local culture [20].

Although existing research provides a certain foundation for understanding new farmers, there are still several shortcomings. First, relevant research is mostly carried out from a single perspective, and there is a lack of comprehensive investigation on the multi-dimensional characteristics of new farmers’ career development. Second, existing research has primarily focused on theoretical analysis or case studies, and there remains a lack of empirical research on the professional competencies of young new farmers and the mechanisms underlying their development. Third, comparative studies between different regions are relatively weak, especially the attention to economically developed areas such as Guangdong is still insufficient. Based on this, this study plans to build an analytical framework for the vocational ability of new farmers, and carry out systematic analysis from three aspects of identity composition, influencing factors and promotion paths, so as to provide empirical support for the career development of young agricultural talents, and also provide reference for the sustainable revitalization of rural areas.

#### (3) Theoretical integration.

Building on existing research on occupational competence frameworks [7,8], this study further integrates relevant theories to broaden the analytical perspective. According to career construction theory, individuals do not passively accept career arrangements; rather, they construct their career development paths and form their career identities through interaction with and continuous adaptation to their environment [21]. This theoretical perspective is helpful to understand how young new farmers with different educational backgrounds enter the agricultural field, construct professional identity and maintain the continuity of career development in the dynamic social and economic situation. At the same time, the concept of career adaptability suggests that individuals primarily rely on the psychological resources of concern, control, curiosity, and confidence when facing career changes and developmental pressures [22]. This theory helps explain how new farmers utilize resources for occupational adaptation to cope with uncertainties in the process of agricultural modernization and translate interdisciplinary knowledge into practical skills. Thirdly, the theory of occupational identity formation provides an important perspective for understanding how new farmers internalize their agricultural role into long-term occupational identity rather than a phased way of making a living, which is consistent with the analytical framework of “entry-stay-development” and can explain the strong occupational identity of this group. Finally, the capability approach further breaks through the limitations of merely looking at issues from the perspective of skills and abilities, emphasizing the real opportunities and external conditions an individual needs to achieve the life goals they value [23]. Under this analytical framework, vocational ability is not only reflected in the knowledge, skills and qualities possessed by individuals, but also embedded in external conditions such as institutional security, resource accessibility and social equity, thereby shedding clearer light on the structural factors affecting the sustainable career development of new farmers.

The above theoretical perspectives suggest that professional competence is not unidimensional; instead, it constitutes a comprehensive system comprising technical ability, adaptive capacity, and practical problem-solving ability. Technical ability is mainly reflected in agricultural production skills and digital literacy. Adaptive capacity includes professional identity and the ability to adjust learning approaches. The actual problem-solving ability focuses on the ability to deal with real problems under limited resources. This is consistent with the analytical logic of “entry – retention – development.” It also helps to integrate the relatively scattered theoretical viewpoints in rural entrepreneurship research.

## 2. Method

Using a multiple-case study approach, this work analyzes the identity construction, influencing factors, and development trajectories of young new farmers. Particular attention is devoted to their performance across the sequential stages of “entry – retention – development.” This paper seeks to systematically examine the key difficulties encountered in their professional competence development and subsequently explores more systematic cultivation pathways and mechanisms. The research results can provide references for optimizing the sustainable development path of rural young people and formulating targeted policies.

From July to August 2023 and July 2024, the research team conducted a questionnaire survey of young new farmers across the province. The questionnaire was developed through a process of design, testing, and revision. The initial set of 25 items was primarily derived from a review of the literature and was validated through pilot testing and field interviews prior to the formal survey. At the same time, the study invited experts and practitioners in the agricultural field to evaluate the clarity, relevance, and completeness of the items, while the interviews focused on competency requirements, skill application scenarios, and real-world issues in sustainable development. After integrating expert opinions and pre-test results, the research team revised and expanded the questionnaire, and finally formed a formal questionnaire with 50 items. The scale is compiled according to the analysis framework of “entry-stay-development” in this study, including four dimensions: professional skills, general skills, core competence and career sustainable development ability, and several specific ability indicators are set under each dimension. All items are rated on a 5-point Likert scale, where 1 indicates “not at all applicable” and 5 indicates “very applicable”; a higher score indicates that the respondent rates their professional competence more highly. A total of 302 valid questionnaires were obtained in the formal survey, and the scale contained 50 items. The reliability test results showed that the Cronbach’s α of the total scale was 0.930, and the α coefficients of the four dimensions were 0.772, 0.724, 0.648, and 0.740, respectively, indicating that the scale as a whole has good internal consistency. The results of validity test showed that the combined reliability (CR) of each dimension was between 0.627 and 0.786, and the average variance extracted (AVE) was between 0.188 and 0.407. The data are suitable for factor analysis. The KMO value is 0.799, and Bartlett’s test of sphericity is significant (p < 0.001). In summary, the scale exhibits sound reliability and validity. It satisfies the fundamental requirements of this study for evaluating the professional competence of young new farmers.

The research protocol was approved by the Ethics Committee of the Science and Technology Office at Guangdong Mechanical and Electrical Polytechnic. This study was conducted in strict compliance with ethical standards. All participants signed an informed consent form before the investigation. Considering the characteristics of peer referral sampling in rural communities, the study particularly emphasizes that participation and referrals are entirely voluntary, refusal will not result in any adverse consequences, and the recommended individuals are contacted directly by the research team to ensure they make independent decisions. The study strictly protects participants’ privacy; questionnaires are anonymized, interview data is pseudonymized and all personally identifiable information is removed, and all data is securely stored and destroyed in accordance with regulations. In addition, participants can withdraw or skip any questions at any time, and interviewers have received appropriate training to minimize the stress that sensitive topics may cause.

Due to the scattered distribution of the research subjects and the diverse types of occupations, traditional random sampling is difficult to implement in practice. Therefore, this study adopts the peer-driven sampling method, leveraging the existing social relationship network to recruit the research subjects. Specifically, the study first invited young new farmers with whom the research team had already established contact to serve as the initial sample for the survey, and asked them to recommend eligible peers from their social circles; the recommended individuals then helped further expand the sample size. This method helps improve the accessibility of the target population, especially suitable for survey subjects who lack a complete directory. However, this approach may also lead to certain deviations, such as an excessive representation of certain groups and a network dependence among the samples, which in turn may affect the generalizability of the results. To minimize this issue, the research further employed K-means clustering analysis. Based on the respondents’ answers in different career ability dimensions, potential types were identified. The elbow method and silhouette coefficient were combined to determine the optimal number of clusters, thereby enhancing the interpretability of the results and the rationality of sample analysis. This study collected 302 valid questionnaires from young and new farmers aged 18–35 in representative counties and towns of Guangdong Province. In order to gain a deeper understanding of their actual situations during the process of career development, the research team also conducted on-site interviews with 10 young new farmers and their respective enterprises in Qingyuan, Jieyang, Meizhou, Heyuan and Shaoguan. The interview outlines covered topics such as motivation for entry, formation of identity, challenges faced in sticking to the agricultural sector, support systems, and future development needs. Before the formal research began, the interview outline was reviewed and revised. Subsequently, the interview data were subjected to thematic analysis based on the questionnaire results to enhance the reliability of the research conclusions.

### 2.1 Implement the steps

#### (1) Seed selection.

When selecting the initial “seed” samples, this study aimed to ensure their representativeness. To this end, the initial participants for this study were mainly identified through the following channels: recommendations from local agricultural departments (such as the Rural Revitalization Bureau), selection of winners from provincial youth agricultural entrepreneurship competitions, and recommendations from influential agricultural cooperatives or agricultural enterprises in various regions. These individuals come from various regions of Guangdong and have diverse professional backgrounds, and to a certain extent, they represent the typical profile of young new farmers.

#### (2) Recruitment and referral.

When recruiting initial participants, the research team will fully explain the purpose of the study, the survey content, the implementation process, and ethical protection measures, with an emphasis on the fact that participation is entirely voluntary and personal information will be strictly confidential, to ensure that they decide whether to participate based on full knowledge. Meanwhile, the research team will provide them with electronic recommendation links so that the subsequent recruitment work can proceed; when these participants recommend other young new farmers, the recommended individuals can also confirm through the corresponding links and enter the survey.

#### (3) Data collection and tracking.

This study collected data through a combination of questionnaire surveys and semi-structured in-depth interviews. The questionnaire part utilized 50 revised questions, mainly for obtaining basic information from the respondents and measuring the professional capabilities of young new farmers within the framework of “entry – staying – development.” The interview section followed a pre-designed outline, focusing on the motivations for entering the profession and the formation of professional identity during the “entry” phase; the challenges faced and support systems available during the “retention” phase; and future plans and growth needs during the “development” phase. On-site interviews were conducted with respondent companies in Qingyuan, Jieyang, Meizhou, Heyuan, and Shaoguan to further understand the specific contexts and influencing factors behind their career development. During the data processing, the research conducted checks on the completeness, logical consistency and value range of the questionnaires; at the same time, the reliability and validity of the formal scale were tested, including indicators such as Cronbach’s α, composite reliability (CR), average variance extracted (AVE), KMO value and Bartlett’s sphericity test. To ensure the transparency and reproducibility of the research process, this paper provides the complete questionnaire items and interview guidelines in the appendix, including the various dimensions, sub-competency indicators, and corresponding guiding questions.

To ensure the transparency of the research process and the reproducibility of the results, this paper attaches the complete questionnaire items and interview outlines used in this study at the end of the article. These include the setting of each dimension, specific sub-ability indicators, and corresponding guiding questions.

### 2.2 Difficulties and solutions

#### (1) Mitigating sample bias.

Since the recruitment of the samples relies on recommendations from social relationship networks, there may be certain sample biases in the study. Regarding this issue, the research team will test the homogeneity of the samples during the data analysis phase; If the sample is found to be overly concentrated or highly homogeneous, we will expand the source pool by adding new initial samples to increase the diversity of the sample.

#### (2) Enhancing referral motivation.

Some initial participants may be unmotivated to remain in the study. Relevant promotional materials were prepared by the research team to explain the practical significance of this study to young farmers, with the aim of enhancing participation. They will provide strong evidence to obtain more policy support and resources.

#### (3) Ensuring data quality.

Respondents may provide inaccurate information when completing questionnaires or participating in interviews. A concise and clear questionnaire was carefully designed in this study to ensure high-quality data, allowing respondents to understand and complete it with ease. The researchers provided appropriate additional guidance during the interviews. This study incorporated essential logic checks and performed data entry reviews to reduce the influence of invalid or inaccurate information. Providing a relatively reliable data foundation for the analysis, the sample of this study covered 21 prefecture-level cities in Guangdong Province, with approximately 300 young new-type farmers surveyed.

## 3. Results and discussion

### 3.1 Identity characteristics and professional identity of young new farmers

The term “new farmers” first appeared frequently in news reports, often in connection with topics such as “innovation and entrepreneurship” and “young people returning to their hometowns.” However, to date, neither the academic community nor society at large has reached a consensus on the specific meaning of this concept.

Based on the data from this study, the research team defined “new farmers” as: New types of agricultural business entities that, in the process of agricultural production and operation, can utilize “Internet + new media” and other means to carry out the production and sales of high-quality agricultural products, and play a role of driving, demonstrating and leading in related practices [24]. Compared to traditional farmers, this group has driven the shift in the concept of “farmer” from a “role-based” to a “profession-based” identity [25,26]. At the same time, they are also an important force in promoting moderately scaled agricultural operations, modern transformation, and rural revitalization [27,28].

The survey data shows that as more and more young people join the new agricultural workforce, the image of this group is being redefined and new characteristics are gradually emerging [29]. Compared with traditional farmers, there is now a noticeable increase in the number of young people among the new generation of farmers who are better educated and have a stronger knowledge base. They pay more attention to ecological agriculture, focus on providing safe and high-quality agricultural products, and strive to increase the added value of agriculture [30]. At the same time, this group usually possesses strong abilities in information acquisition, technology application, and agricultural industrialization management [31].

The survey results presented in Table 1 more clearly reflect the demographic characteristics and professional identity status of young new farmers in Guangdong Province. This group is predominantly young in terms of age structure. Individuals aged 19–26 account for 61.11%. The proportion of women is 39.19%, indicating that women have gradually become an important force in rural revitalization. With respect to education level, associate degree or higher was held by 87.42% of respondents, among whom bachelor’s and master’s degree holders made up 19.54%. Overall, young farmers in Guangdong Province generally have the characteristics of being relatively young in age and having a high level of education.

**Table 1:** Demographic, educational, and occupational characteristics of the survey sample.

Demographics	Category	N	Percentage (%)
Gender	Male	184	60.81
	Female	118	39.19
Age	14-18 years	42	13.89
	19-26 years	184	61.11
	27-35 years	21	6.94
	>35 years	55	18.06
Educational background	High school or below	38	12.5
	College degree	205	68.06
	Bachelor degree	38	12.5
	Master degree	21	6.94
Professional identity	Recognize young new farming as a profession	284	94
	Do not recognize young new farming as a profession	18	6
Career prospects	Not very optimistic	6	2
	Generally optimistic	12	4
	Relatively optimistic	48	16
	Very optimistic	236	78
Engagement intention	As a long-term career	256	84.67
	As a short-term career	34	11.33
	Other options	12	4

Meanwhile, this group demonstrates a strong sense of professional identity and holds a relatively positive attitude toward their career prospects. Table 1 shows that 94% of the respondents regarded “young farmers” as a profession rather than merely an entrepreneurial form. Most respondents held a “very optimistic” or “relatively optimistic” attitude towards the prospects of this profession, and 84.67% of the respondents expressed their willingness to choose it as a long-term career. Further chi-square test results indicate that there are significant differences in the distribution of the sample across gender, age, and educational level: there are significantly more males than females (χ² = 14.42, p < 0.001), ages are mainly concentrated in the 19–26 age range (χ² = 217.86, p < 0.001), and most respondents have a junior college education (χ² = 301.16, p < 0.001). Furthermore, the aspects of professional identity (χ² = 500.85, p < 0.001), career prospect assessment (χ² = 468.60, p < 0.001), and long-term career intention (χ² = 361.93, p < 0.001) also clearly favored the positive options. As shown in the figure, young farmers are not only young and highly educated, but they also generally exhibit a strong sense of professional identity, a high degree of confidence in their future prospects, and a strong desire to continue in this profession.

This strong sense of professional identity and positive expectations are closely related to their upbringing in the era of mobile internet. Compared to the previous generation of professionals, they are not only more familiar with the internet and new media technologies, but also more open to new business models such as e-commerce and live streaming [32]. In addition, with a certain emotional connection to agriculture and rural life itself, and supported by relevant national policies, many young people are more willing to return to their hometowns to explore sustainable agricultural development paths. Therefore, at the policy level, in addition to providing resource support, it is also necessary to further strengthen the guidance on career identity, so as to help this group achieve their long-term agricultural development goals more stably.

### 3.2 Factors influencing the development of the young new farmers group

#### 3.2.1 Internal motivations.

**Fig 1:**
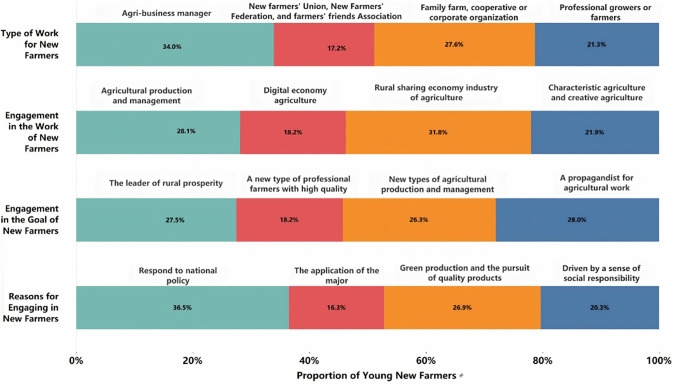
Survey of young new farmers’ work motivation.

Analysis of survey data on work motivations and professional roles (Fig 1) reveals key internal drivers and characteristics of young new farmers:

(1) Diverse professional backgrounds before engaging in agriculture

The background of young new farmers is no longer limited to traditional agricultural workers; instead, their professional backgrounds have become much more diverse. As shown in Fig 7, 34.0% of respondents are mainly engaged in agricultural management work, 21.3% are professional growers and livestock farmers, 27.6% work in organizations such as family farms, cooperatives, or enterprises, and 17.2% participate in informal organizations such as new farmer alliances. The results of the chi-square test indicate that there is a significant difference in this distribution (χ² = 19.83, p < 0.001), with agricultural enterprise managers accounting for the highest proportion. This suggests that, in the course of modern agricultural development, an increasing number of individuals with management and entrepreneurial skills are entering this field.

**Fig 2:**
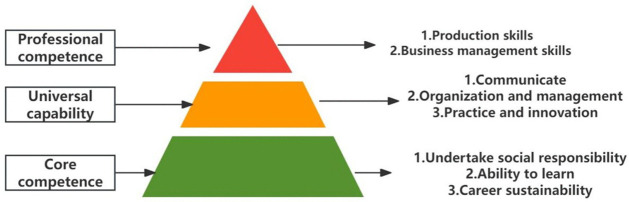
Occupational ability iceberg structure model.

**Fig 3:**
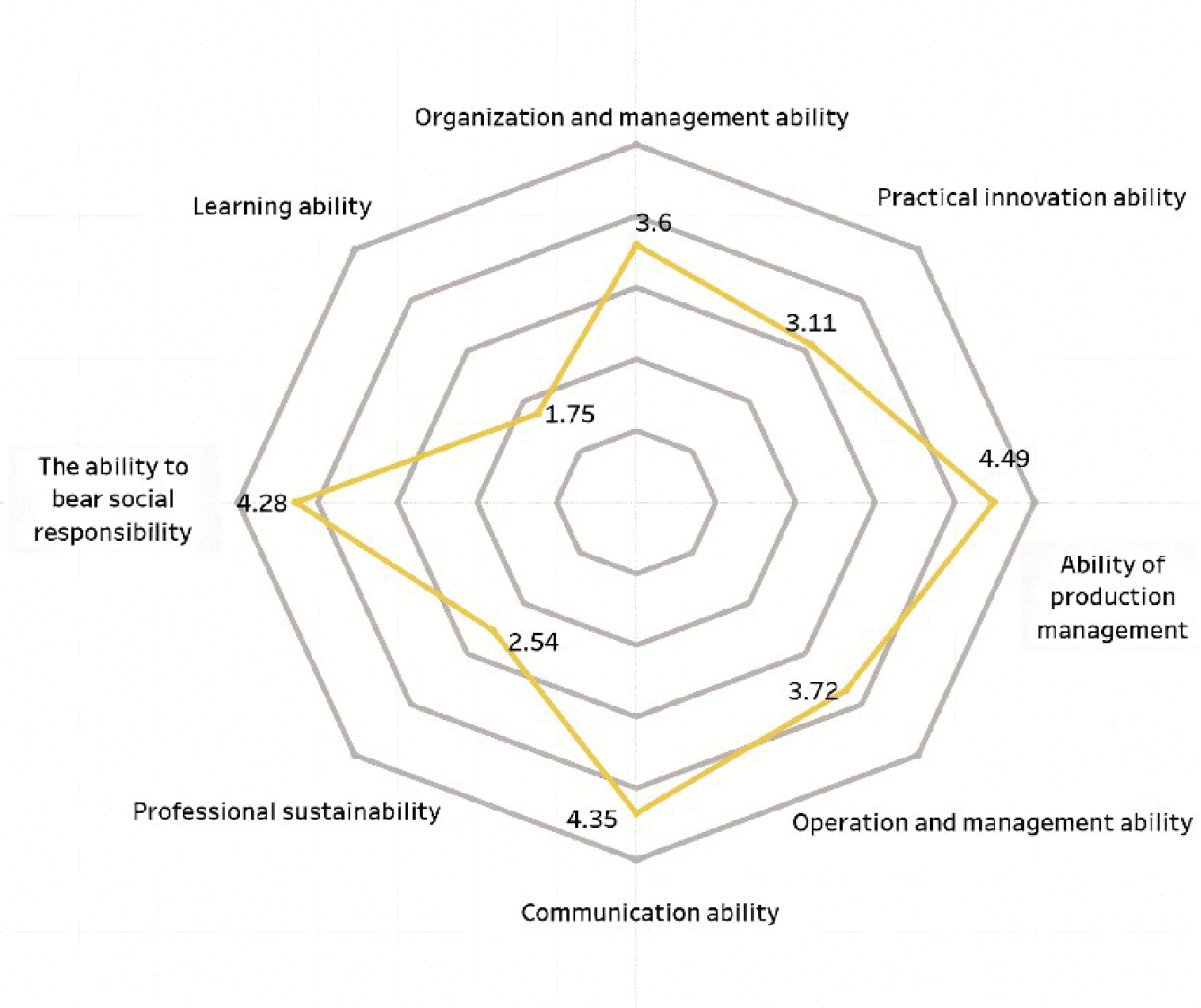
Scored chart of vocational ability in science and engineering for survey sample.

**Fig 4:**
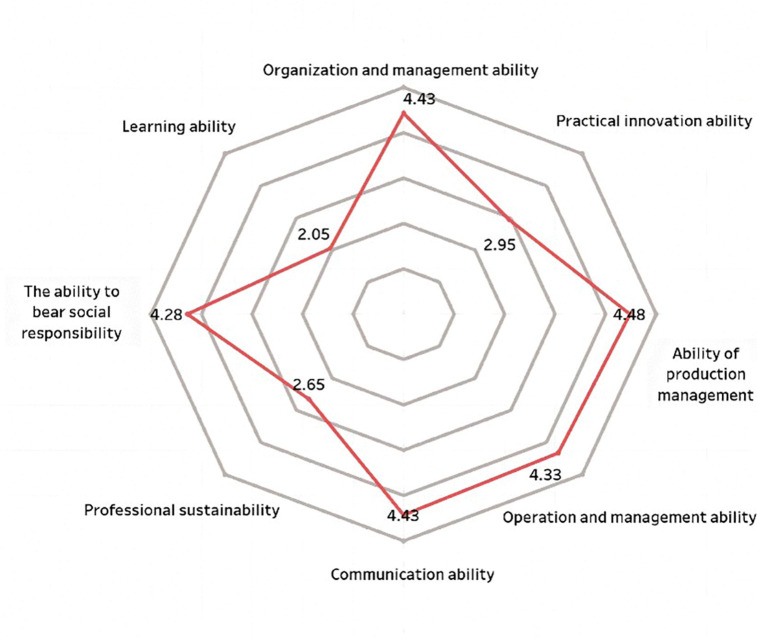
Scored chart of vocational ability in economics and management for survey sample.

**Fig 5:**
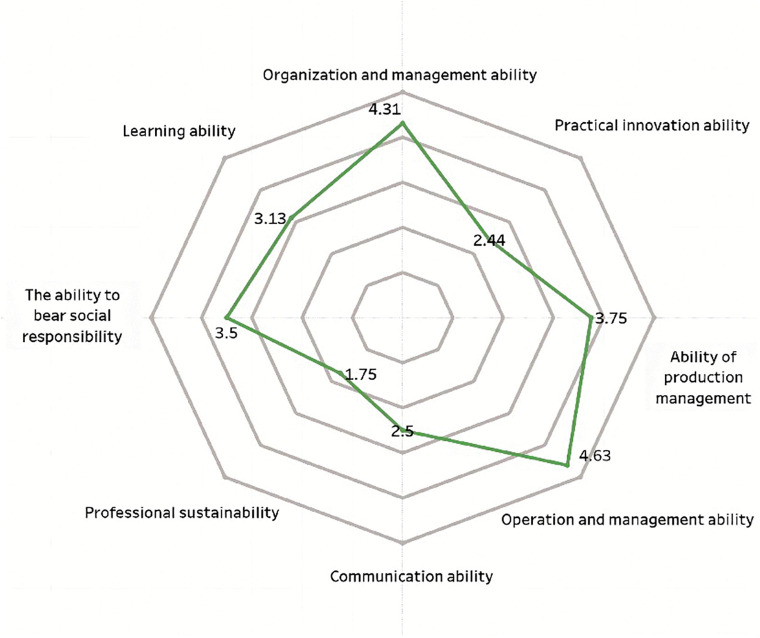
Scored chart of vocational ability in humanities for survey sample.

**Fig 6:**
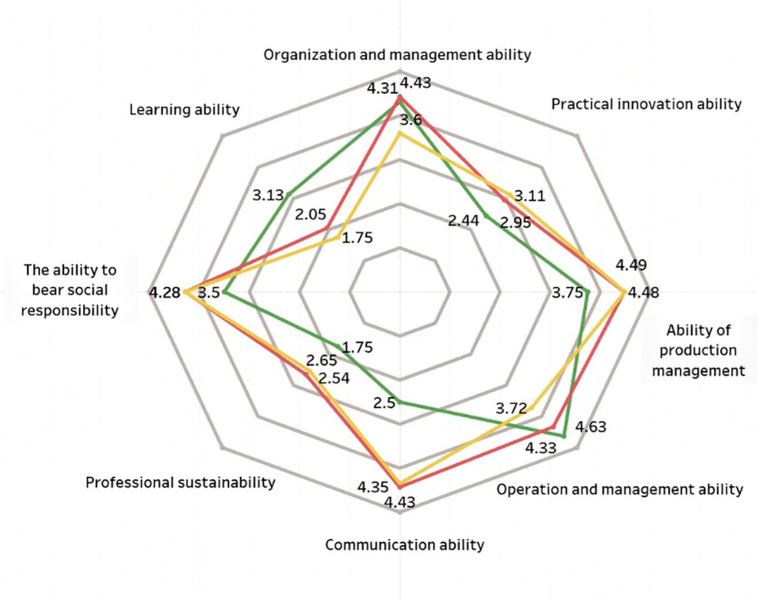
Comparison of scores in three major categories of vocational ability among survey samples.

In terms of background, young new farmers can generally be divided into four categories: those who are well-educated, people who have switched from other industries to engage in agriculture, returning migrant workers, and local rural residents. Due to their different backgrounds, there are certain differences among them in terms of their employability and entrepreneurial skills, their ability to adapt to new environments, and their approaches to resource integration.

(2) Diverse work engagements in agriculture

**Fig 7:**
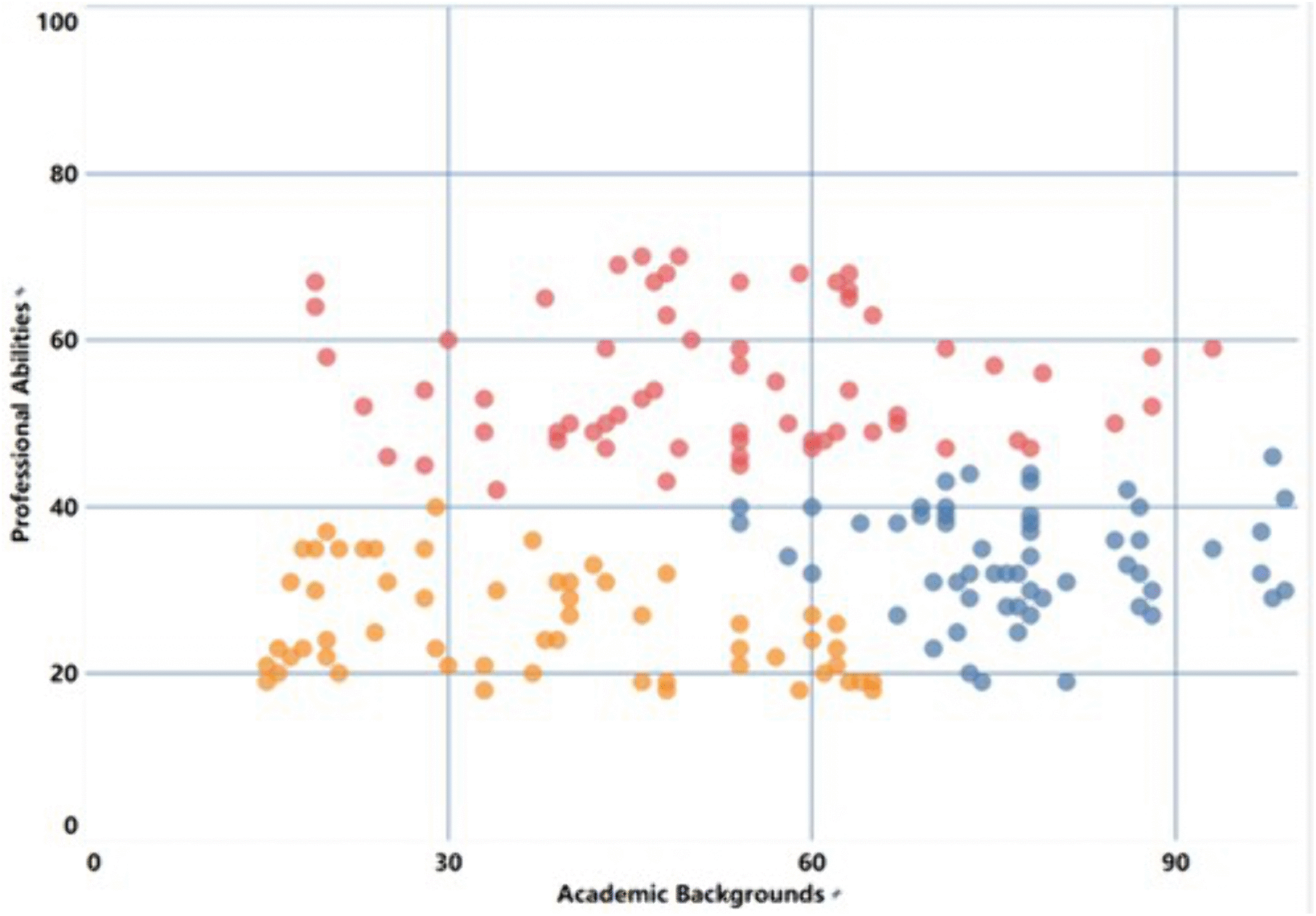
Survey sample clustering based on professional abilities using the k-means model.

The career paths of young new farmers are diverse. As shown in Fig 7, 31.8% of people are engaged in the rural sharing economy, 28.1% are involved in agricultural production and management, 21.9% are engaged in specialty or creative agriculture, and 18.2% are involved in digital agriculture. The results of the chi-square test indicate that this distribution difference is statistically significant (χ² = 13.52, p = 0.004). This suggests that, although young new farmers are active in various fields, the rural sharing economy remains the primary employment direction, and also reflects that digital technology and new business models are accelerating their entry into the agricultural sector. In addition to directly engaging in agricultural production and operation, they are also actively involved in various exchange activities such as forums, markets, training sessions and competitions. They also actively use technological tools, e-commerce platforms and social media for business operations, demonstrating strong characteristics of modern agricultural development.

(3) Emergence of new professional identities and mindsets

From the perspective of professional role perception, young new farmers exhibit a relatively diverse self-positioning. According to Fig 7, 28.0% consider themselves as agricultural advocates, 27.5% believe they are the drivers of rural revitalization, 26.3% consider themselves as innovators in production and operation, and 18.2% position themselves as high-quality professionals. The results of the chi-square test indicate that this difference in distribution was not statistically significant (χ² = 7.67, p = 0.053), suggesting that there is no clear hierarchy among the various types of role identification. In other words, although young new farmers have a strong sense of identity with this occupational group, they are not consistent in specific development directions and role choices, reflecting obvious diversification characteristics.

(4) Key motivations for engaging in farming

The motivation analysis reveals that young new farmers’ entry into the agricultural sector is influenced by the external policy environment as well as closely related to their personal development aspirations. According to Fig 7, the primary reason for engaging in agriculture is to respond to national policies, accounting for 36.5%; the second is the recognition of the concepts of green production and high-quality products, accounting for 26.9%; those motivated by a sense of social responsibility account for 20.3%; and those hoping to leverage their professional expertise account for 16.3%. The results of the chi-square test indicate that there are significant differences among the various types of motivation (χ² = 28.34, p < 0.001), suggesting that policy factors remain the primary reason for attracting young people to the agricultural sector.

#### 3.2.2 Educational background and professional competency tendencies of young new farmers.

Table 2 shows that the educational backgrounds of young new farmers exhibit distinct non-agricultural characteristics; those who have received formal agricultural education are in the minority, while the majority come from a variety of academic disciplines. Among them, the proportion of backgrounds in economic management and science and engineering is relatively high, which is quite consistent with the current trend of agricultural production and operation continuously transforming toward digitalization and market orientation. Their diverse professional backgrounds give this group a distinct advantage in information acquisition, business management, and technology application, and provide a talent pool for the development of new models such as “agriculture plus the Internet.” From this, it can be seen that young new farmers are no longer the traditional image of farmers. Instead, they more prominently represent a new type of agricultural practitioners who possess a comprehensive knowledge background, management skills and technical awareness.

**Table 2:** Educational background, academic disciplines, and professional competency tendencies of the survey sample.

Demographics	Category	N	Percentage (%)
Educational background	Related to agriculture	50	16.44
	Involved in agriculture	87	28.77
	No agricultural background	165	54.79
Academic disciplines	Agronomy	37	12.33
	Science and engineering	106	35.62
	Literature or law	40	13.7
	Economics or management	112	36.99
	Medical science	4	1.37
Digital Technology Acceptance	Very unacceptable	8	2.74
	Unacceptable	25	8.22
	Moderately acceptable	58	19.18
	Acceptable	132	43.84
	Highly acceptable	79	26.03
Digital Platform Proficiency	Very unacceptable	8	2.74
	Unacceptable	12	4.11
	Moderately acceptable	74	24.66
	Acceptable	108	35.62
	Highly acceptable	100	32.88

As shown in Table 2, only 16.44% of young new farmers have an educational background directly related to agriculture, 28.77% have an indirectly related background, and 54.79% have no agricultural background. The results of the chi-square analysis indicate that this distribution difference is statistically significant (χ² = 68.47, p < 0.001), suggesting that the sources of personnel entering the agricultural sector are relatively diverse, and individuals from different professional backgrounds are continuously entering this field.

From the perspective of subject distribution, different professional backgrounds show significant differences. Table 2 shows that the economics or management major accounts for 36.99%, the science or engineering major accounts for 35.62%, the literature or law major accounts for 13.70%, the agriculture major accounts for 12.33%, and the medicine major accounts for only 1.37%. The results of the chi-square test indicate that this difference in distribution is statistically significant (χ² = 149.56, p < 0.001), suggesting that the disciplinary composition of the sample is not balanced, with economics and management, as well as science and engineering, accounting for a higher proportion. This kind of professional structure is conducive to introducing digital technology, management methods, and new business models into the agricultural sector, but it also indicates that some new farmers still lack traditional agricultural knowledge and practical experience.

Meanwhile, Table 2 also reveals the strong professional characteristics of this group as well as their adaptability to digital technologies. Specifically, 69.87% of young new farmers are open to modern technologies such as the internet, big data, blockchain, and artificial intelligence, with 26.03% indicating a high level of acceptance. The results of the chi-square test indicate that this difference in distribution is statistically significant (χ² = 156.91, p < 0.001). This suggests that, overall, this group holds a relatively positive attitude toward the use of technology. In addition, Table 2 shows that a considerable number of respondents are proficient in using e-commerce platforms, social media, mobile payment applications, and smart devices. Of these, 32.88% reported having a high level of proficiency. The results of the chi-square test indicate that this distribution difference is also statistically significant (χ² = 149.98, p < 0.001), indicating that proficiency in using digital platforms is generally at a high level.

At present, young new farmers are gradually integrating mainstream e-commerce and social media platforms (such as Douyin, Weibo, and Taobao) into their daily operations. At the same time, they have begun using automated equipment such as drones, sensors, and robots, as well as smart services like data analytics and cloud computing, to improve production efficiency and supply chain management. This indicates that they have become an important force in promoting the transformation and upgrading of traditional agriculture. However, the rapid pace of technological updates also brings new pressures to agricultural operations, especially for small-scale operators who are more likely to encounter difficulties in the adaptation process. Therefore, future policy support should focus more on helping small-scale farmers alleviate problems such as high costs of technology investment and difficulties in actual operation.

### 3.3 Factors influencing group development from a vocational ability perspective

#### 3.3.1 Composition of new farmers’ vocational ability.

The structure of an individual’s professional capabilities is in correspondence with their professional knowledge system. According to relevant theoretical models [33], both can be divided into three interrelated levels: professional knowledge comprises foundational knowledge, basic professional knowledge, and specialized professional knowledge; while occupational competencies include core employability skills, industry-specific competencies, and job-specific competencies. Based on this framework, and combined with interviews and questionnaires, this paper analyzes the understanding of the composition of the professional competencies of new farmers. In the specific assessment, the Iceberg Model (see Fig 2) is used to classify professional competencies, with the surface layer being professional skills, the middle layer being general skills, and the deep layer being core competencies.

The professional capabilities of new farmers mainly consist of three aspects: First, professional capabilities, which refer to the abilities in production and operation as well as management; second, general capabilities, including communication and coordination, organizational management, and practical innovation capabilities; third, core competencies, namely, social responsibility, learning ability, and sustainable career development ability [7]. This study uses a five-point rating scale to compare new farmers from different disciplinary backgrounds, including science and engineering, economics and management, and the humanities. A higher score indicates that their competency structure is more closely aligned with the needs of modern agricultural development. As shown in Figs 3–5, there are significant differences across disciplinary backgrounds. This not only reflects the varying educational focuses of different disciplines but also highlights the important role played by interdisciplinary and multidisciplinary professionals in the development of agricultural innovation. Therefore, in terms of policy making and talent cultivation, more attention should be paid to interdisciplinary training to promote the improvement of the comprehensive abilities of new farmers.

Fig 3 shows that new farmers with backgrounds in science and engineering scored higher in production management skills (4.49) and business management capabilities (3.72), indicating that they possess strong technical application skills in modern agricultural production. The score for social responsibility is 4.28, which is also at a relatively high level. In contrast, their communication skills score is only 2.54, significantly lower than the scores of other competency dimensions, indicating that they are relatively weak in interpersonal communication and collaboration. The results of the variance analysis showed that there was a statistically significant difference in communication skills among those with different academic backgrounds (F = 6.27, p < 0.01). Among them, the participants with a science and engineering background scored the lowest. New farmers with a background in science and engineering have certain advantages in terms of technical expertise and social responsibility, but they still need to improve their communication, coordination, and collaboration skills. These abilities are crucial to agricultural growth.

New farmers with backgrounds in economics and management scored highest in organizational management skills (4.43) and communication skills (4.43) (see Fig 4), demonstrating their strengths in business operations, strategic planning, and market expansion. They scored lower in learning ability (2.05) and career sustainability (2.65), which means they excel in coping with the business environment but may face challenges in continuous learning and long-term career development. Further statistical analysis revealed that their average sustainability score was significantly lower than that of other groups (F = 5.84, p < 0.01). This suggests that while they have strengths in management, they still lack lasting adaptability. This discovery suggests that, based on the existing management expertise, efforts should be focused on enhancing adaptability and the level of sustainable development.

Fig 5 shows that new farmers with a background in the humanities scored higher in organizational management skills (4.31) and business management skills (4.63), indicating that they have certain advantages in organizational coordination and business operations. However, the scores in terms of professional sustainable development ability (1.75) and practical innovation ability (2.44) were relatively low, indicating that there are still deficiencies in their long-term career development and the application of innovation in agricultural practices. The analysis of variance results showed that the differences in practical innovation ability among different academic backgrounds are statistically significant (F = 7.12, p < 0.01), with the group from humanities backgrounds experiencing more noticeable adaptation pressure. Overall, this group performs well in organizational coordination and business management, but still needs to further improve in sustainable development and innovative practices.

A comparative analysis shows that new farmers with different academic backgrounds exhibit significant differences in their capabilities. Those with backgrounds in science and engineering tend to have stronger technical skills, but there is still room for improvement in communication and market management; Those with an economic management background tend to perform better in leadership coordination and business operations, but they have relatively weaker technical adaptability; those with a humanities background have advantages in organizational management, but they still need to further enhance their capabilities related to agricultural production. These differences indicate that talent cultivation aimed at the needs of modern agricultural development should place greater emphasis on the integration of interdisciplinary abilities, promoting the coordinated enhancement of technology, management, and innovation capabilities.

Fig 6 shows that new agricultural professionals with different academic backgrounds exhibit distinct characteristics in terms of professional competencies. Those with backgrounds in economics and management scored highest in organizational and communication skills, both at 4.43, but scored relatively lower in learning ability (2.05) and sustainability skills (2.65). Those with a background in science and engineering perform stronger in production management, with a score of 4.49, but their communication skills score lower, only 2.54. Those with a background in the humanities score the highest in business management ability, at 4.63, but are relatively lacking in sustainable development ability (1.75) and innovation ability (2.44). The results of the variance analysis showed that there were significant differences in communication skills, learning abilities, and innovation capabilities among different academic backgrounds (all p < 0.01), while the differences in technical abilities among different groups were relatively small. Overall, new farmers with different academic backgrounds have their own strengths and weaknesses. In the future, targeted ability training should be carried out based on their academic characteristics to promote the comprehensive improvement of their professional abilities.

Overall, new farmers from different academic backgrounds each have their own distinct characteristics. Individuals with a background in science and engineering tend to have well-rounded skills and strong practical and innovative abilities, but their communication skills are relatively weak; those with a background in economics and management have clear strengths in production, operations, and organizational management; and those with a background in the humanities perform well in policy comprehension, brand awareness, and communication and coordination, but their overall capabilities are relatively weaker. This indicates that more targeted support and training should be provided based on different academic backgrounds.

#### 3.3.2 Portrait identification of the young new farmer group.

**Fig 8:**
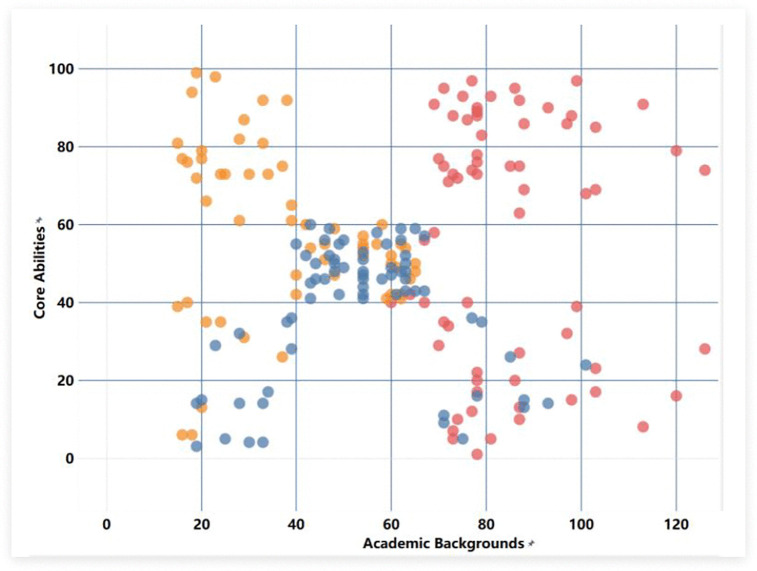
Survey sample clustering based on core abilities using the k-means model.

**Fig 9:**
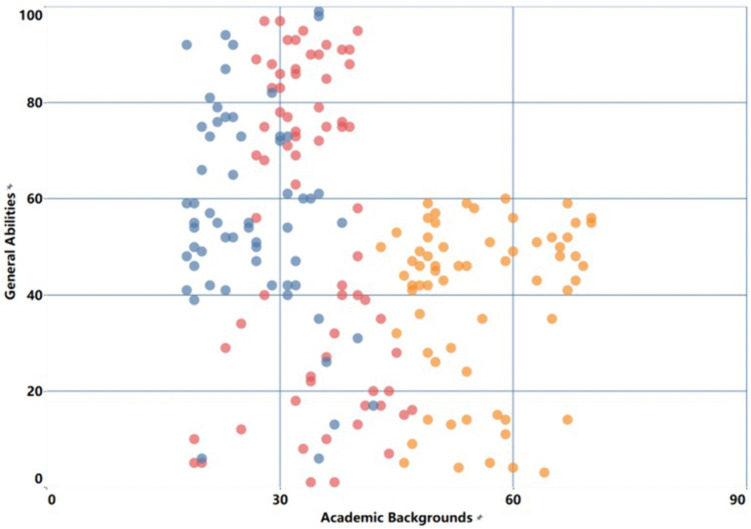
Survey sample clustering based on universal abilities using the k-means model.

New farmers with different professional backgrounds show certain differences in general abilities, professional abilities and core abilities. Among them, science and technology-related fields, economic management fields and humanities fields are represented by yellow, red and blue respectively. Quantitative analysis based on survey data, combined with an improved K-means clustering method [34], this study classified and assessed the professional capabilities of young new farmers, thereby establishing a more scientific capability profile for them and providing a more targeted basis for subsequent training and development. Figs 7–9 further illustrate the results of occupational competency assessments and cluster analysis across different disciplinary contexts.

After completing data preprocessing, this paper selected nine dimensions under the three secondary indicators— “Professional Competence,” “Core Competence,” and “General Competence”—as input variables, set the maximum number of algorithm iterations to 10, and presented the format of the preprocessed data. To facilitate comparison with existing quantitative evaluation results, the data for each dimension was first categorized and then calculated using predetermined weights. Since these data all come from the nine dimensions of the capacity structure of young new farmers, an optimized intelligent algorithm was further used to perform classification analysis, and dimensionality reduction and clustering were completed. Using professional variables as the basis for case division, the number of clusters K was set to 3. The results show that the distances between the cluster centers are relatively large, and the minimum spacing setting is reasonable, indicating that the clustering effect is good. Each cluster center formed in the end can be regarded as a representative of a group of ability characteristics, and the data distributed near this center reflects the typical ability level of that group.

Fig 7 illustrates the relationship between academic background and professional competence. As professional competence increases, the distinctions between different groups become more pronounced, and there is less overlap. This indicates that new farmers with different academic backgrounds possess varying levels of foundational knowledge in areas such as production management and business administration; therefore, their training methods should be tailored to their specific backgrounds in order to better enhance their capabilities in these areas. Fig 8 shows the clustering of core competencies. Compared with other dimensions, the aggregation degree of core competencies is higher, indicating that the consistency among the indicators within this competency module is relatively strong. In other words, new farmers from different disciplinary backgrounds can adopt relatively similar training approaches when improving their core competencies. Fig 9 presents the classification results of general abilities. Among them, there is a certain overlap between the economic management group and the humanities background group, indicating that these two groups have similarities in the improvement of general abilities and can adopt a relatively consistent improvement path. In contrast, the characteristics of the science and technology background group are more prominent, and targeted training needs to be carried out based on their own situations [35].

### 3.4 Discussion

The research findings indicate that while young new farmers possess strong learning abilities and good overall qualities, there is still room for improvement in terms of talent recruitment, education and training, and targeted support mechanisms. This study is consistent with the conclusions of existing research, namely, that rural young professionals, although possessing high development potential, still face many limitations in terms of access to resources and guidance support [36]. At the same time, this study further found that, in the context of Guangdong Province, the development of young new farmers depends not only on individual capabilities but is also influenced by regional socioeconomic conditions and the policy environment, thereby expanding the scope of discussion in related research to some extent [37].

First of all, the attractiveness of this field still needs to be enhanced. Although the entry threshold is relatively low, many young people still lack understanding of the development opportunities within it. This is consistent with the perspective of career adaptability theory, which holds that career concern and career curiosity are important prerequisites for individuals to discover future opportunities and enter new career paths [38]. However, from a practical perspective, although this field has considerable room for growth, many young people still have not truly recognized the opportunities within it [37,39]. This finding also underscores once again that a lack of career awareness remains a significant factor hindering rural development [39]. The government and educational institutions should further strengthen career guidance and relevant publicity efforts in order to address the above issues. Through new media communication, exemplary models, and the cultivation of social responsibility, more outstanding young people can be attracted to engage in rural entrepreneurship and agricultural innovation [2,40]. However, this study has certain regional limitations, and the applicability of its conclusions remains limited. In the future, similar studies could be conducted in other provinces or rural areas to further compare the differences in relevant strategies across different cultural and economic contexts [41,42].

Second, educational and training programs for young new farmers need to be made more targeted. Research findings indicate that there are significant differences in competency levels among respondents with different academic backgrounds. Those with backgrounds in economics and management perform better in strategic decision-making and business management, while graduates from science and engineering disciplines tend to be relatively weaker in communication-related skills [43], those with a background in humanities and social sciences generally lack strong entrepreneurial motivation [44]. This result is basically consistent with the findings of existing research, that is, disciplinary background affects individuals’ professional competence and career adaptability [38]. Further examination reveals that this study also found that the diversity of academic backgrounds can also affect the way young people adapt to the roles of rural revitalization. Different educational experiences and professional training may have different effects on career aspirations, preparation levels, and adaptation to development [45]. From the perspective of occupational adaptation and the formation of professional identity, this suggests that successful entry into the rural workforce depends on the interaction between individual resources and external support [46,47]. Therefore, universities and training institutions should design differentiated training programs by combining the strengths and weaknesses of different academic backgrounds [44].

Thirdly, the relevant support mechanisms need to be further enhanced in terms of precision and development. Research data shows that the majority of young new farmers mainly undertake management responsibilities in agricultural operations, and their work focuses on production organization and business operations. This indicates that the demand for talent in rural revitalization is no longer limited to a single skill, but increasingly requires compound talents who possess management skills, technical abilities, and practical coordination capabilities [48], this finding is also consistent with the conclusions of recent studies on the transformation of rural human capital [49]. The capability-oriented theory provides an important perspective for understanding this phenomenon. It emphasizes that the continuous development of a career not only depends on individual capabilities, but is also influenced by structural conditions such as policy support, social security, and public services [50]. Therefore, subsequent support should not remain at the level of general assistance, but should pay more attention to training and development by discipline and stage, especially strengthening support in production management, business strategy, and technology application [51].

The interpretation of the results of this study should remain cautious. As the data comes from self-reports in the Guangdong region, the conclusions of the study may be affected by subjective response biases and regional contextual differences [52]. Furthermore, although this study has revealed differences in skill structures and support needs, the roles played by motivation, rural identity, and psychological adjustment in long-term rural engagement have not yet been fully explored [53,54]. Future research could combine qualitative interviews, multi-source data, or longitudinal research designs to further investigate these issues. Overall, based on existing studies, this research not only deepens our understanding of the new young farmers in Guangdong, but also provides a more targeted analytical perspective for understanding young people’s participation in rural revitalization.

## 4. Conclusions and recommendations

Previous research on new farmers has largely adopted specific perspectives, with a focus on aspects such as the use of digital technology, entrepreneurial identity [55]. The findings of this study should be interpreted with caution. As the data are based on self-reports from Guangdong Province, the conclusions may be subject to self-response bias and regional differences. Important references for understanding the new farmer group are provided by these studies. Nevertheless, they fail to adequately examine either the comprehensive makeup of new-type farmers’ professional competence or issues related to sustainable development, instead tending toward descriptive accounts from relatively limited perspectives.

Offering a new analytical perspective for related research, this study is grounded in the “entry—retention—development” pathway. It integrates theories related to career adaptability, career identity formation and abilities, demonstrating the multi-level characteristics of career abilities. It also explains how the new generation of young farmers adapt to the agricultural environment, confirm their career positioning, and seek more promising development opportunities. The significance of this study also lies in incorporating these findings into broader discussions on career development, vocational education, and rural transformation.

This study focuses on the main difficulties and development potential that young new farmers encounter during their growth process. Considering the actual conditions and feasibility of implementation, four targeted policy suggestions are further proposed, aiming to provide support for high-quality talent cultivation and rural revitalization.

First, industrial upgrading should not be limited to general supply chain expansion; it should also provide clear pathways for young people to enter the agricultural sector. Survey results show that more than 60% of respondents are between the ages of 19 and 26, and are primarily engaged in new business models such as rural e-commerce and agritourism (see Figs 2 and 7). Many respondents also mentioned that the lack of systematic industry pilot projects is an important factor restricting entrepreneurship. Therefore, grassroots governments can promote pilot projects targeting young people in accordance with actual conditions, such as agricultural technology incubation platforms, local e-commerce support policies, and the construction of characteristic agricultural tourism clusters [56]. Of course, this process will also face practical challenges such as land-use restrictions and high initial investment costs, while also requiring attention to the risk of saturation in popular niche sectors. By rolling out pilot programs in phases and conducting ongoing monitoring and evaluation, we can help attract more young talent to rural areas and alleviate the problem of talent drain from rural to urban areas.

Second, capacity-building efforts should focus on the skill gaps that have already emerged among young new farmers. Research has found significant differences in competency levels across different academic backgrounds: science graduates scored higher in technical and production skills but scored relatively lower in communication skills (2.54 points); liberal arts graduates performed better in coordination skills but scored the lowest in innovation and sustainability. Based on this situation, universities, especially non-agricultural ones, should set more targeted training content according to the actual needs of different groups, such as strengthening communication and leadership training for science students, and supplementing courses in entrepreneurship and business management for liberal arts graduates [57]. The survey results also indicate that the respondents have a strong demand for digital skills. Specifically, 67.16% of the respondents stated that they possess the ability to use e-commerce and online tools. This suggests that it is necessary to further carry out digital skills enhancement training and establish a guidance mechanism between graduates and local successful entrepreneurs. During the implementation process, issues such as continuous funding and mismatch between skills supply and demand may arise, but they can be alleviated through strengthened cooperation between industry and academia [58].

Third, various resources should be further integrated to create opportunities for young new farmers to exchange knowledge and gain practical experience. Many respondents mentioned difficulties in adapting to the local environment after leaving their hometowns. The survey results also showed that nearly half of the respondents had no prior background in agriculture (Fig 8). This situation highlights the necessity of establishing a more systematic and practical training platform. For example, semester-long rural internships integrated with local projects, innovative practical activities organized jointly by schools and businesses, and formal mentorship networks involving experienced farmers [59]. During the implementation process, it is necessary to consider how to maintain the continuous participation of all parties and how to control costs. However, these issues can be resolved by establishing appropriate subsidies, creating incentive mechanisms, and clarifying the responsibilities of all parties [60]. By standardizing and institutionalizing such practical platforms, young new type farmers can better integrate into the rural development process.

At last, the improvement of the service system should be personalized to address young people’s practical concerns about the sustainable growth of rural areas. Many interviewees cited inadequate childcare, healthcare, and job security as key factors affecting their decision to settle in rural areas. For one, the results show that the lack of access to childcare services has a more pronounced impact on young female farmers. Women account for 39% of the sample (see Fig 1). To meet these practical needs, local governments can further improve rural collaborative childcare services, expand the coverage of mobile medical and elderly care services, and provide more centralized and comprehensive service windows for young entrepreneurs [61]. It is also necessary to attach importance to the establishment of a social security system that is compatible with flexible agricultural employment, and to provide convenient channels for resolving disputes. It should be noted that the implementation of these measures will still be constrained by financial capacity and pressures on grassroots governance. Therefore, a more prudent approach would be to implement the project in stages, with priority given to conducting pilot projects in areas where young talents are concentrated It is also necessary to continuously assess the effectiveness of the relevant policies to avoid imposing additional burdens on the lives of existing residents [62].

Building upon a comprehensive analysis of survey data and interview materials, this study establishes a more practical and operational analytical framework designed to support the sustainable development of young new farmers. The measures proposed in this article, such as industrial guidance, capacity building, practical training and service support, are conducive to deepening our understanding of issues related to vocational abilities, and also provide a reference framework for rural revitalization-related practices.

## Supporting information

S1 Appendix A Questionnaire items. (DOCX)

S2 Appendix B Semi-structured interview protocol. (DOCX)
