# Supplementary material for: Development status of young new farmers from the perspective of vocational ability: A research analysis based on 21 cities in Guangdong Province
Source: PLoS One. 2026 May 22;21(5):e0349224. doi: 10.1371/journal.pone.0349224 (PMC13196931; doi:10.1371/journal.pone.0349224)
Supplement: S1 Appendix — (DOCX) [file pone.0349224.s001.docx]

**Appendix A. Questionnaire Items**

**I. Professional Competence**

1. Basic Knowledge and Skills

1. I have basic agronomic knowledge (e.g., cultivation, breeding, pest control).
2. I can operate common agricultural tools and machinery.
3. I understand basic agricultural laws and regulations.

2. Production Management Skills

1. I can allocate land, labor, and equipment effectively.
2. I can use modern equipment (e.g., drones, sensors) in farming.
3. I can adopt environmentally friendly farming methods (for instance, reducing the use of chemical fertilizers).

3. Business Management Skills

1. I am able to analyze market demand and choose appropriate products.
2. I can devise marketing strategies and establish distribution channels.
3. I can use modern management methods to operate farms or cooperatives.

**II. General Competence**

1. Communication and Coordination Ability

1. I collaborate with my colleagues and share experiences.
2. I consulted the government department about policies and sought support.
3. I maintain effective communication within organizations.

2. Organizational Management Ability

1. I can plan the development direction and goals of an organization.
2. I can hire and assign staff as needed.
3. I can organize online and offline agricultural activities.

3. Innovative Practice Ability

1. I use the internet and new media to build agricultural brands.
2. I can use new marketing models (such as live stream sales).
3. I can combine agriculture with tourism or the cultural industry.

**III. Core Competence**

1. Social Responsibility

1. I value honesty, dedication, and rural revitalization.
2. Working in agriculture gives me a sense of pride and fulfillment.
3. I am committed to green production and high-quality products.

2. Learning Ability

1. I am able to manage my time and set clear learning goals.
2. I take the initiative to learn agricultural knowledge by myself.
3. I use online platforms to learn new things.

3. Professionalism

1. I have received professional and practical training.
2. I am able to adapt to social changes and pressures.
3. I have set clear career goals and development plans.

**IV. Vocational Sustainability**

1. I have clear career preferences and goals.
2. I continuously learn through training and continuing education.
3. I have practical agricultural experience and technical skills.
4. I am able to integrate various resources to improve the efficiency of the farm.
5. I can collect and analyze agricultural policies and information.
6. I am paying attention to the new trends in agricultural development.
7. I can translate teamwork and experience into business growth.
8. I am able to obtain resources that support my career development (land, funds, policies).
