# Supplementary material for: Development status of young new farmers from the perspective of vocational ability: A research analysis based on 21 cities in Guangdong Province
Source: PLoS One. 2026 May 22;21(5):e0349224. doi: 10.1371/journal.pone.0349224 (PMC13196931; doi:10.1371/journal.pone.0349224)
Supplement: S2 Appendix — (DOCX) [file pone.0349224.s002.docx]

Appendix B. Semi-Structured Interview Protocol

**Purpose**

This interview seeks to explore the career paths of the new generation of young farmers in the context of rural vitalization. With the participants’ permission, the interviews were recorded in full and used for research purposes only, safeguarding the anonymity and confidentiality of the participants.

**Questions:**

1. How long have you held your current position? What are your main responsibilities?
2. Which industries do you think have the greatest demand for young talent? Do the skills of returning overseas students align with local needs?
3. What policies has the government enacted to attract and support young people returning to their hometowns? What are the strengths and weaknesses of each of these policies?
4. What measures do you think help young people grow, and how effective are they?
5. What challenges are involved in attracting and retaining young talented individuals? What are the underlying causes of these difficulties?
6. Why do young people come back? What makes them stay? And why might they leave again?
7. What activities and training programs are offered for young returnees?
8. Do you think there are any public platforms where young people can interact with one another?
9. How would you rate the public services, social security, and environmental improvements in rural areas?
10. How is the progress of local industrial development? Has it provided diverse opportunities?
